# *Campylobacter jejuni* and *Campylobacter coli* from Houseflies in Commercial Turkey Farms Are Frequently Resistant to Multiple Antimicrobials and Exhibit Pronounced Genotypic Diversity

**DOI:** 10.3390/pathogens12020230

**Published:** 2023-02-01

**Authors:** Hannah Bolinger, William G. Miller, Jason A. Osborne, Jeffrey Niedermeyer, Sophia Kathariou

**Affiliations:** 1 Department of Food, Bioprocessing, and Nutrition Sciences, North Carolina State University, Raleigh, NC 27695, USA; 2 Produce Safety and Microbiology Research Unit, Agricultural Research Service, US Department of Agriculture, Albany, CA 94710, USA; 3 Department of Statistics, North Carolina State University, Raleigh, NC 27695, USA

**Keywords:** *Campylobacter*, turkeys, flies, antimicrobial resistance, multidrug resistance, multilocus sequence typing, clonal diversity

## Abstract

*Campylobacter* is a leading foodborne pathogen, and poultry are a major vehicle for infection. Houseflies play important roles in colonization of broiler flocks with *Campylobacter* but comparable information for turkey farms is limited. Here, we investigated houseflies as potential vectors for *Campylobacter* in 28 commercial turkey flocks. We characterized species, genotypes, and the antimicrobial resistance (AMR) profiles of *Campylobacter* from turkey feces and houseflies in the same turkey house. Of the 28 flocks, 25 yielded *Campylobacter* from turkey droppings and houseflies, with an average of 6.25 and 3.11 *Campylobacter* log CFU/g feces and log CFU/fly, respectively. Three flocks were negative for *Campylobacter* both in turkey feces and in houseflies. Both *C. coli* and *C. jejuni* were detected in turkey feces and houseflies, with *C. coli* more likely to be recovered from houseflies than feces. Determination of *Campylobacter* species, genotypes, and AMR profiles revealed up to six different strains in houseflies from a single house, including multidrug-resistant strains. For the predominant strain types, presence in houseflies was predictive of presence in feces, and vice versa. These findings suggest that houseflies may serve as vehicles for dissemination of *Campylobacter*, including multidrug-resistant strains, within a turkey house, and potentially between different turkey houses and farms in the same region.

## 1. Introduction

*Campylobacter jejuni* and *C. coli* are major contributors to human foodborne disease worldwide [1,2,3,4,5]. *Campylobacter* infections are typically self-limiting, with symptoms including diarrhea (often bloody), fever, and abdominal cramps, but infection can also lead to severe sequelae such as Guillain–Barré Syndrome [6]. Antimicrobial treatment may be indicated for certain cases of human campylobacteriosis, with the drugs of choice being macrolides and quinolones [7]. Drug-resistant *Campylobacter* has been designated as a serious antibiotic resistance threat by the Centers for Disease Control and Prevention (CDC), requiring “prompt and sustained action” [8]. Leading vehicles for human campylobacteriosis include poultry, raw milk, and untreated water [9,10,11,12]. *Campylobacter* is often recovered from broiler and turkey flocks, and colonization can reach up to 10^9^ CFU/g cecal content [13,14,15,16,17].

Reducing the number of *Campylobacter*-positive poultry flocks may significantly reduce human illness; therefore, there is significant interest in producing *Campylobacter*-free poultry [18,19,20]: however, this can be difficult as the routes of introduction into poultry flocks are poorly understood. Insects such as houseflies and darkling beetles have been recognized as possible vectors for the introduction of *Campylobacter* into a flock, with most work focusing on flies [21,22,23,24]. Several studies have investigated fly-mediated transmission of *Campylobacter,* and *Campylobacter* spp. have been isolated from flies in commercial broiler farms [25,26]. Additionally, *C. jejuni* was shown to be transferable to flies from *Campylobacter*-positive broilers, and from these flies to previously *Campylobacter*-negative chickens [27].

Houseflies are known to travel several miles, and thus may serve as vectors of transmission between farms [28,29]. Studies in Denmark found that at least 900 flies entered the broiler house every day, and a UK study was able to collect approximately 31 flies every two hours [30]. Fly screens greatly reduced the number of flies entering the houses, and also significantly reduced the prevalence of *Campylobacter*-positive flocks at slaughter [31]. However, limited information is available on the genotypes and antimicrobial resistance of fly-derived strains [25,30,32]. Furthermore, the available reports on *Campylobacter* and flies have focused on broilers, mostly in Europe and the UK, with a noticeable dearth of data on turkeys, for which the United States is the world’s leading producer [33]. Such data are needed, considering that turkeys have been shown to be commonly colonized with *C. jejuni* and *C. coli*, including multidrug-resistant strains [13,15,16,17,34]. The objective of the current study was to investigate the prevalence, antimicrobial resistance, and strain types of *Campylobacter* from turkey feces in commercial turkey farms, and from flies in the same turkey houses.

## 2. Materials and Methods

### 2.1. Sample Collection and Campylobacter Isolation

Turkey feces and flies from 28 distinct houses on 23 farms in eastern North Carolina were investigated over three years (2013–2015). On certain occasions, different flocks were grown at the same farm during different seasons. As is common practice for intensive, conventional turkey production in the United States, young turkeys (“brooders”) were kept on brooder farms from day-of-hatch to four or five weeks of age. At that point, the brooder flocks were transported to multiple “growout” farms, where they remained until marketing. The 28 flocks investigated in this study were derived from 15 brooder and 8 growout farms, and flock age ranged from 11 days to 17 weeks. Turkey fecal droppings and flies were collected from the same turkey house for each flock. To collect the fecal samples, the poultry house was divided into quadrants, and three fecal droppings were collected from each quadrant, yielding a total of 12 fecal samples / house. Freshly voided fecal droppings were collected using sterile swabs, placed into sterile 15-mL polypropylene tubes (Corning, Corning, NY, USA), and transported to the laboratory on ice. During the same visit, up to 10 live flies from the same turkey house were captured in Ziploc bags, and were transported to the laboratory on ice. Fecal samples and flies were typically processed for *Campylobacter* within the same day.

To process the fecal samples, six fecal samples (0.1 g each) from two quadrants were combined in 6 mL of sterile Mueller–Hinton broth (MHB; Difco, Becton Dickinson, Franklin Lakes, NJ, USA), to make a fecal suspension yielding two composite samples/flock. Serial dilutions of these suspensions were plated onto modified charcoal-cefoperazone-deoxycholate agar (mCCDA; Oxoid, Hampshire, UK; SE 155, Oxoid), and were incubated microaerobically at 42 °C for 48 h in anaerobic jars containing CampyPak Plus microaerobic sachets (Becton Dickinson). A total of six putative *Campylobacter* colonies per composite sample were picked based on colony morphology on mCCDA and purified in Mueller–Hinton agar (MHA; Difco, Becton Dickinson). Pure cultures were preserved at −80 °C in brain heart infusion broth (Becton Dickinson) supplemented with 20% glycerol. To isolate and enumerate *Campylobacter* from the flies, each fly was homogenized in 1 mL of sterile phosphate-buffered saline, and 100 μL of the suspension was plated on mCCDA. Subsequent incubations and purifications were as described for the fecal samples.

### 2.2. Characterization of Campylobacter Isolated from Turkey Feces and Flies

Multiplex PCR, using primers targeting *ceuE* and *hipO*, was used to determine the species (*C. coli* or *C. jejuni*, respectively) of the isolates, as described previously [15]. Susceptibility to a panel of antimicrobials was determined as previously described [35]. The tested antimicrobials and resistance breakpoints included ≥ 16 μg/mL tetracycline (T), ≥ 64 μg/mL streptomycin (S), ≥ 8 μg/mL erythromycin (E), ≥ 64 μg/mL kanamycin (K), ≥ 50 μg/mL gentamicin (G), and the quinolones ≥ 32 μg/mL nalidixic acid (N) and ≥ 4 μg/mL ciprofloxacin (C), with resistance to both quinolones designated Q. Each antimicrobial susceptibility determination included the pansensitive *C. jejuni* strain ATCC 33560 for quality assurance, as described [35]. Isolate designations consisted of the combination of species designation and antimicrobial resistance (AMR) profile: for example, *C. jejuni* resistant to tetracycline (T), kanamycin (K), and gentamicin (G) would be designated *C. jejuni* TKG. A panel of isolates which represented different species and AMR profile combinations were characterized by multilocus sequence typing (MLST). Genomic DNA was extracted from cultures grown on MHA using the Qiagen DNeasy Blood and Tissue Kit (Qiagen, Valencia, CA, USA) following the procedures suggested by the vendor and DNA was eluted with 100–200 μL of AE buffer provided with the kit. The genomic DNA was used for MLST as previously described [36]. Minimum spanning trees (MST) were constructed as previously described [36], and were employed to visualize relationships among the different MLST-derived sequence types (STs).

### 2.3. Statistical Analysis

Analysis of data was carried out using various procedures in the SAS statistical software package (SAS Institute, Inc., Cary, NC, USA). Relative abundance of *C. jejuni* and *C. coli* was compared across sources (feces, flies) using chi-square statistics from two-way contingency tables. Additionally, a generalized linear mixed model for the probability that an observed isolate was *C. coli* was fitted, using the GLIMMIX procedure of SAS: this model included fixed source effects and random farm effects, to account for the fact that the counts populating the contingency table arose from repeated measurements on some of the farms. To quantify diversity, the ratio of the number of different species–AMR profile combinations found to the total number of isolates was calculated for each farm and source (feces, flies). These ratios were modeled non-parametrically with additive sample type and farm effects, and Friedman’s test was used to test for sample type effects.

A manual search of the absolute frequencies identified strains found in only one sample type. Fisher’s Exact test was used to investigate the source effect on the genotype (ST). To assess similarity between strains from flies and feces, a two-by-two table of frequencies was constructed for fly-and-feces-positivity: for example, of the 25 *Campylobacter*-positive flocks, 14 were found to be positive for strain *C. jejuni* TSKQG in both feces and flies, while five flocks were negative for this strain in both the flies and feces; one flock was positive for *C. jejuni* TSKQG in flies but not feces, while the remaining five flocks were *C. jejuni* TSKQG-positive in feces but not flies. Fischer’s Exact Test was used to test the hypothesis that the presence of a strain in one sample type was not associated with its presence in the other sample type from that same flock.

## 3. Results

### 3.1. Campylobacter Co-Occurred in Turkey Feces and Houseflies in the Turkey Houses

Of the 28 flocks, 25 were *Campylobacter*-positive, with an average 6.25 ± 0.74 log CFU/g feces, while *Campylobacter* could not be recovered from the turkey droppings from three flocks: these three flocks were from the same farm but from different production cycles spanning more than one year (August 2013, November 2013, and December 2014).

The flies from the turkey houses were all identified as houseflies (*Musca domestica*), upon consultation with Dr. Wesley Watson, entomologist at North Carolina State University. Analysis of the houseflies (hereafter referred to as “flies”) from the turkey houses revealed complete correlation in *Campylobacter*-positive status between turkey feces and flies; all flocks with *Campylobacter*-positive birds were in turkey houses with flies that were also positive for *Campylobacter*, while *Campylobacter* could not be recovered from any of the flies captured in the turkey houses with *Campylobacter*-negative birds. In no case was a flock positive in just one source, i.e., only feces or only flies.

A total of 254 flies were captured and analyzed for *Campylobacter*, with an average of 9.0 flies/turkey house. Of the 254 captured flies, 60.5% +/− 31.9% were positive for *Campylobacter*, with positivity per turkey house visit ranging between 0 and 100%, and with average *Campylobacter* populations of 3.11 ± 0.98 log CFU/*Campylobacter*-positive fly. In all, 380 *Campylobacter* isolates (164 from flies and 216 from feces) were characterized from the 25 *Campylobacter*-positive flocks. The isolates were nearly evenly split between *C. coli* (*n* = 192) and *C. jejuni* (*n* = 188) (Table 1). Table 1 shows the number of *C. jejuni* and *C. coli* isolates from each source in each year of the study. 

### 3.2. Flies Tended to Harbor More C. coli than C. jejuni and More Diverse Campylobacter Strains than Feces

As indicated above, *C. jejuni* and *C. coli* contributed similarly (*n* = 188 and *n* = 192, respectively) to the total *Campylobacter* spp. (*n* = 380) characterized from turkey feces and flies in the turkey farms (Table 1). However, the relative proportions of *C. jejuni* and *C. coli* differed between feces and flies. While *C. coli* and *C. jejuni* were isolated with similar frequencies from turkey fecal droppings (48.6% and 51.4%, respectively), *C. coli* was isolated more frequently from flies compared to *C. jejuni* (Table 1). A generalized linear mixed model indicated that *Campylobacter* isolated from flies was more likely to be *C. coli* than when isolated from feces (*p* < 0.0001), with a mean probability of a feces or fly-derived isolate being *C. coli* of 0.3776 and 0.6967, respectively.

**Figure 1:**
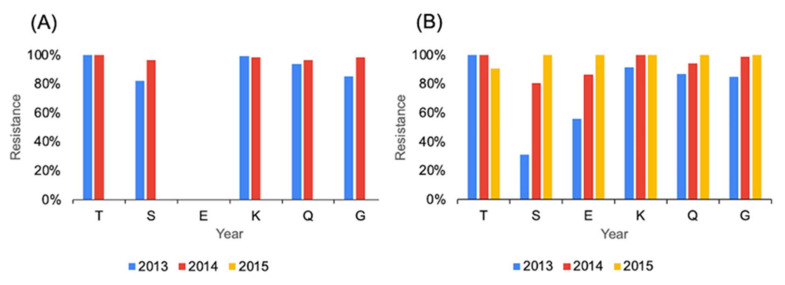
Antimicrobial resistance of (**A**) *C. jejuni* and (**B**) *C. coli* during each year of the study. T, S, E, K, and G indicate resistance to tetracycline, streptomycin, erythromycin, kanamycin, and gentamicin, respectively, while Q indicates resistance to both nalidixic acid and ciprofloxacin. Resistance was determined as described in Materials and Methods.

The prevalence of *C. coli* resistant to certain antibiotics increased each year from 2013 to 2015, with this trend being especially noticeable for streptomycin (S) and erythromycin (E) (Figure 1); however, resistance to the other tested antimicrobials was present in ≥ 85% of isolates in all years. Some increases were also noted between 2013 and 2014 in the prevalence of *C. jejuni* with resistance to streptomycin and gentamicin (Figure 1). *C. jejuni* was not obtained in 2015, most likely due to the small number of samples collected (Table 1). Additionally, no *C. jejuni* isolates were resistant to erythromycin, but the percentage of *C. jejuni* resistant to all other tested antimicrobials was consistently high (> 80%) during 2013 and 2014 (Figure 1).

**Figure 2:**
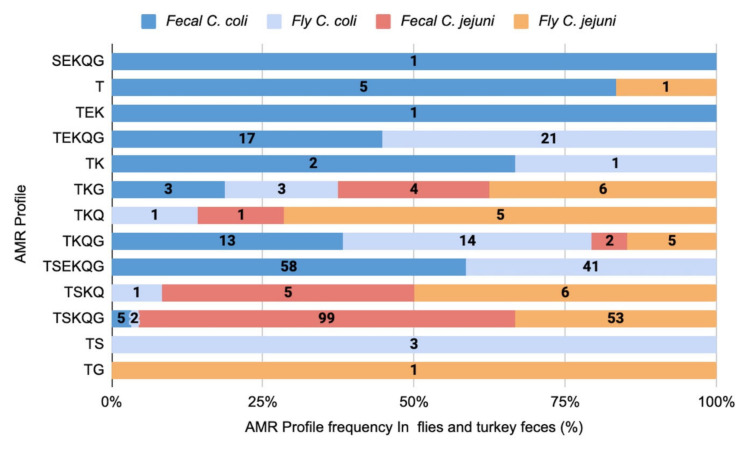
Relative contribution of each sample type (feces and flies) to the AMR profiles noted in *C. jejuni* and *C. coli*.

Seven distinct AMR profiles were detected among fly-derived *C. jejuni*, and five were detected in *C. jejuni* from the feces (Figure 2). Except for a single isolate from feces that was only resistant to tetracycline, *C. jejuni* exhibited resistance to at least two antimicrobial classes (Figure 2). A single AMR profile, i.e., the multidrug-resistance profile TSKQG, clearly predominated in *C. jejuni* regardless of source, even though it was more dominant among feces-derived *C. jejuni* (89%) than among *C. jejuni* from flies (69%) (Figure 2).

Among *C. coli* isolates, there was an equal number (*n* = 9) of AMR profiles in both sample sources (Figure 2). With the exception of a small number (*n* = 5) of *C. coli* from feces that were resistant only to tetracycline, all other isolates were resistant to two or more antimicrobial classes (Figure 2). The most common AMR profile in both feces and fly-derived *C. coli* was TSEKQG, followed by TEKQG and TKQG (Figure 2). However, despite the similarities in the predominant AMR profiles between the two sources, AMR profile diversity was greater (*p* < 0.01) among fly-derived isolates (0.5002) than among those from feces (*p* = 0.2773).

### 3.3. Certain Campylobacter Species–AMR Profile Combinations Exhibit Significant Predictive Potential for Turkey Feces vs. Fly Origin of the Campylobacter Isolates

Fisher’s Exact test was employed to determine the predictive potential of detecting specific combinations of *Campylobacter* species and AMR profiles across the two sources, i.e., turkey feces and flies in the turkey house. The detection of several dominant species–AMR profile combinations in flies from a turkey house was found to have predictive potential for the occurrence of the same combination in feces from the same turkey house, and vice versa: for example, detecting *C. jejuni* TSKQG in feces could successfully predict that flies in the same turkey house harbored *C. jejuni* TSKQG (*p* = 0.0068), and vice versa. Significant predictive potential was also found for the *C. coli* with the leading AMR profile, TSEKQG (*p* = 0.0119), and to a lesser extent, for *C. coli* TKQG (*p* = 0.0235), *C. coli* TEKQG (*p* = 0.0333), and *C. coli* TK (*p* = 0.0400).

### 3.4. C. jejuni and C. coli Genotypes Are Largely Shared between Flies and Feces, but Certain C. coli Genotypes May Be More Likely to Be Encountered in Flies

**Figure 3:**
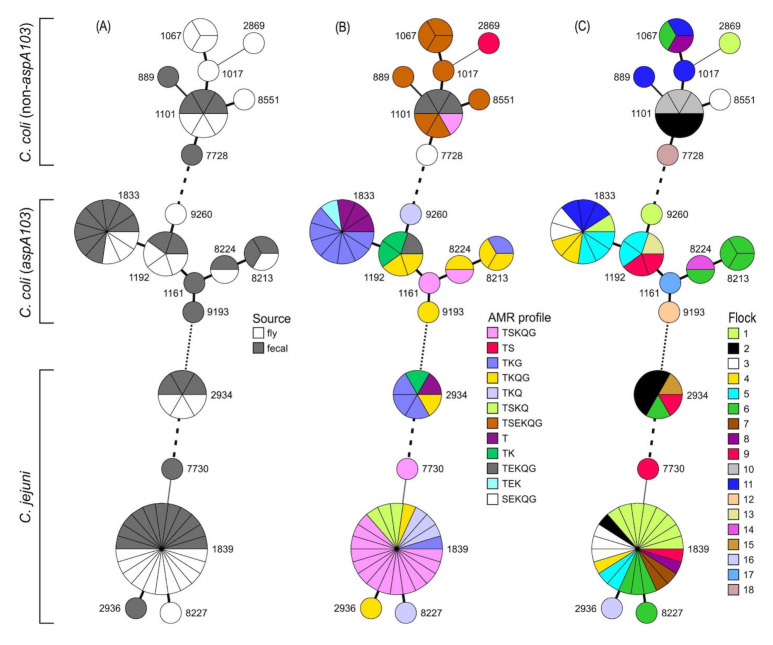
Minimum spanning tree depicting the clustering of 19 identified STs by (**A**) source, (**B**) AMR profile, and (**C**) flock. The tree was created as previously described [36], with each ST represented by a circle, and each segment within the circle corresponding to an individual isolate. Thick, short lines connect single-locus variants, while longer, thin lines connect double-locus variants; black, dashed lines indicate three or more allele differences, and the stippled line between *C. jejuni* and *C. coli* indicates five or more allele differences. The two clusters of *C. coli*, one consisting of all *aspA103* isolates, and the other of the remaining *C. coli* isolates, are indicated. The isolates are listed in Supplemental Data Table S1.

A panel of 69 isolates (31 *C. jejuni* and 38 *C. coli*) were chosen for genotyping by MLST. The panel included 36 isolates from feces and 33 from flies, representing different AMR profiles, and derived from 18 flocks from 17 turkey farms (Supplemental Data Table S1). MLST genotyping identified five different STs for *C. jejuni*, and 14 for *C. coli* (Figure 3A). Eight novel STs were identified, including two among *C. jejuni* (STs 8227 and 7730) and six among *C. coli* (STs 8213, 8224, 9260, 9193, 7728, and 8551). Two distinct clusters were noted in *C. jejuni*, one consisting of ST-2934, and the other of the remaining *C. jejuni* STs (Figure 3A). *C. coli* STs were also partitioned into two major clusters, one of which included all STs with the *aspA103* allele, which is normally typical of *C. jejuni* (Figure 3A). The *C. coli* ST cluster with the *aspA103* allele had been identified before in the genotypic analysis of *C. coli* from diverse sources, and had been referred to as “Cluster A” [36]. Most of the isolates in the *C. coli aspA103* cluster in the current study were susceptible to erythromycin, in contrast to the other cluster, which included the majority of erythromycin-resistant *C. coli* isolates (Figure 3B).

*Campylobacter* isolates with the same AMR profiles tended to exhibit the same or related STs (Figure 3B). For example, in *C. jejuni* the TSKQG AMR profile was found only in ST-1839 and the related ST-7730 (Figure 3B). In *C. coli*, the AMR profile TSEKQG was associated with five closely-related STs with one or two allele differences (STs 889, 1101, 1017, 1067, and 8551), with ST-1067 being the most common (Figure 3B). One exception was *C. coli* with the AMR profile TEKQG, which was detected among isolates with the distantly-related STs 1101 and 1192 (Figure 3B).

In *C. jejuni*, the predominant ST among isolates from both feces and flies was ST-1839 (Figure 3A), encountered in multiple turkey farms (Figure 3C). ST-1839 isolates were multidrug resistant, and most had the dominant AMR profile TSKQG, but TSKQ, TKQ, TKG, and TKQG were encountered as well (Figure 3B). Only four other STs were encountered among *C. jejuni* from feces or flies, with three STs encountered in only one isolate (two in feces, and one in a fly), and the third (ST-2934) in both feces and fly-derived isolates (Figure 3A). STs encountered in three or more isolates were consistently shared between turkey feces and flies (Figure 3A).

A more even distribution of different STs was noted among *C. coli*. Two STs (STs 1101 and 1833) were most common in *C. coli*, but they did not overwhelmingly dominate the population in the way noted with ST-1839 in *C. jejuni*. Five of the 14 *C. coli* STs were encountered in three or more isolates (Figure 3A). Four of these (STs 1101, 1833, 1192, and 8213) were detected in *C. coli* from feces as well as flies, while ST-1067 was encountered only in *C. coli* from flies (Figure 3A) from different flocks (Figure 3C). Interestingly, ST-1192 and ST-1067 were closely related (one or two allele differences) to ST-1833 and ST-1101, respectively, which were encountered among both fly and feces-derived *C. coli* (Figure 3A), suggesting the potential for strain diversification in flies. 

### 3.5. Associations between Genotypes and AMR Profiles in C. jejuni and C. coli from Turkey Feces vs. Flies

As described above, ST-1839 was the predominant ST in both flies and feces (Figure 3A). All fly-derived *C. jejuni* isolates of ST-1839 (*n* = 11) had the predominant AMR profile TSKQG (Figure 3B). However, *C. jejuni* ST-1839 from turkey feces (*n* = 11) exhibited four AMR profiles in addition to TSKQG (*n* = 3) (Figure 3B).

In *C. coli*, two STs (STs 1101 and 1833) were repeatedly (*n* = 6 and 11, respectively) encountered among the 38 *C. coli* isolates that were genotyped (Figure 3A). For *C. coli* with ST-1101, two AMR profiles (TSEKQG and TSKQG) were identified among the fly-derived isolates, while all isolates from feces had a different MDR profile, TEKQG (Figure 3B). In the case of ST-1833, all three isolates from flies had the AMR profile TKG, while two additional profiles were encountered (T and TEK) (Figure 3B) among the eight ST-1833 isolates from feces.

Of the remaining five STs with three or more isolates, four (STs 1101, 1833, 8213, and 1192) were found in both feces and flies (Figure 3A), as described above. Interestingly, of the five *C. coli* isolates with ST-1192, two flocks, each with two isolates, showed the same AMR profiles (Figure 3B,C), suggesting that ST-1192 isolates from the same farm shared the same AMR profile. Also of note, the AMR profiles shown by isolates with ST-1192 had the MDR profiles TSKQG, TKQG, and TEKQG, which did not overlap with those of ST-1833, the closest (one-allele difference) ST (Figure 3B). Only one of the flocks was in common between ST-1833 and ST-1192 isolates (Figure 3C).

## 4. Discussion

In this study, *Campylobacter* isolates from turkey feces and flies in the turkey farms were characterized via a combination of their species designations, AMR profiles, and MLST-based genotypes. Few reports are available describing the genotypes of *C. jejuni* and *C. coli* from flies in livestock farms, e.g., cattle [37,38], sheep [38], broilers [25], and turkeys [39]. Furthermore, there is a dearth of information on the AMR profiles in the fly-derived isolates, and their relatedness to those from the animals. Thus, the current study provides the first glimpse into the major *C. jejuni* and *C. coli* STs, as well as AMR profiles circulating in turkeys and flies in turkey farms in the United States.

We were able to isolate *Campylobacter* at high levels from the turkey feces of all but 3 of the 28 flocks in the study. The three flocks from which *Campylobacter* could not be recovered from the turkeys were grown during different, non-overlapping time periods at the same farm, suggesting that this particular farm was consistently negative for *Campylobacter*. The underlying reasons were not determined; however, based on our observations and records, this farm appeared to enforce more rigid biosecurity practices than other farms in the study [40].

There was significant agreement between the prevalence of *Campylobacter* in the turkey feces and carriage in the flies, as in no case was a turkey house positive only for flies or only for feces; furthermore, most of the *C. jejuni* and *C. coli* STs encountered in two or more isolates were shared between the turkey feces and the flies. Genotypic relatedness between fly-derived *Campylobacter* strains and strains from the associated food animals were noted previously with broilers and cattle [25,38], and were also noted in another study with two turkey farms in Italy [39], though to a lesser extent than in the current study.

We noted a high prevalence of *Campylobacter*-positive flies in our study (60.5%), and a similarly high prevalence (66.7%) was noted in the above-mentioned study of two turkey farms in Italy [39]. *Campylobacter* was detected via PCR, and was also shown to be viable in 13.4% of the flies from two turkey farms in Arkansas, USA [41]. On the other hand, an earlier investigation in Norway failed to detect *Campylobacter* in a turkey farm, while 43.2% and 50.7% of the flies from a pig farm and a chicken farm, respectively, were positive for *C. jejuni* [26]. The high prevalence (approx. 66–67%) of *C. jejuni* and *C. coli* in the flies from turkey houses in our study and the study by Piccirillo et al. [39] is in contrast to the lower values noted for flies in broiler farms, which ranged from undetectable [42] to 8.2% [25]. Relatively low prevalence of *Campylobacter* (8.9%) was also noted in a cattle farm [37] and a farm with both cattle and sheep (5.8%) [38], while flies from another cattle farm were negative for *Campylobacter* [26]. The concentration of *Campylobacter* in animal feces is expected to be critical in determining the prevalence of *Campylobacter*-positive flies, and the high levels of *Campylobacter* that were noted in this study in the turkey feces (6.25 ± 0.74 log CFU/g) are likely to have been a major contributor to the high prevalence of *Campylobacter*-positive flies. Temperature, time, and dose may affect the survival of *Campylobacter* in flies [43]. Higher temperatures negatively affect the survival of *Campylobacter* in the fly but also allow increased propagation of houseflies [32,43].

Persistence of *Campylobacter* in flies may be limited, with limited or no recovery of *Campylobacter* after 24 h at temperatures exceeding 25 °C [43,44]: however, these studies utilized experimental oral inoculation of the flies with laboratory-grown *C. jejuni* strains, and there may be important strain-to-strain variation in persistence. Furthermore, *C. jejuni* or *C. coli* naturally present in the poultry houses may have different fates in the flies than laboratory-grown strains, especially in the presence of the flies’ natural microbiota. *Campylobacter* populations in the flies were not enumerated in the previous studies of turkeys or other food animals. The relatively high levels of *Campylobacter* in the *Campylobacter*-positive flies (3.11 ± 0.98 log CFU/*Campylobacter*-positive fly) likely reflected the high levels in the feces, and variation in the levels among flies from the same *Campylobacter*-positive turkey house may reflect time between feeding and capture, as well as dosage.

We found that *C. coli* was noticeably more genetically diverse than *C. jejuni*, regardless of whether it was derived from feces or flies. Higher diversity in *C. coli* from turkeys was also reported in a previous study of three turkey farms in Ohio [45], while the opposite was found with two turkey farms in Italy [39]. The underlying reasons remain to be elucidated, and may reflect regional differences in the *C. jejuni* and *C. coli* strains colonizing turkey flocks. Regional influences were also suggested in the study of strains from Italy [39], as well as by our findings with *C. jejuni* ST-1839, which appears to be predominant in turkeys in North Carolina according to the current study and previously [34,35,46], while not encountered in isolates from turkey farms in Ohio [45]. In fact, of the 22 STs detected in the turkey farms in Ohio [45], only three—the *C. coli* STs 889 and 1017, and the *C. jejuni* ST-2934—were shared with those in our study, and none of the STs in the current study were shared with those from the turkey farms in Italy [39].

While the prevalence of the two species was nearly evenly split between *C. coli* and *C. jejuni*, we found that *C. coli* was statistically more likely to be found in flies and *C. jejuni* more likely to be found in feces. *C. jejuni* can survive at least as well as *C. coli* in environmental samples [47,48,49,50]; however, it may be that *C. jejuni* strains predominating in the turkeys and excreted in the feces may have lower relative survival outside the GI tract than similarly excreted *C. coli*, and thus lower likelihood of being acquired by the flies. It is also possible that *C. coli* may survive longer in the flies than *C. jejuni*, thus being more likely to be isolated from the flies.

The dominant *Campylobacter* STs in the turkey feces, i.e., the *C. jejuni* ST-1839 and the *C. coli* STs 1101 and 1833, were also those most commonly isolated from flies. The *C. jejuni* isolates of ST-1839 were all multidrug resistant and showed resistance to every antimicrobial in the panel with the exception of erythromycin, likely reflecting the fitness costs of erythromycin resistance in *C. jejuni* [51]. Surprisingly, however, the fly-derived *C. jejuni* isolates of ST-1839 had the predominant AMR profile TSKQG, while those from the turkeys exhibited several additional AMR profiles. Thus, even though ST-1839 was dominant in both flies and feces, the flies tended to preferentially harbor ST-1839 with one specific multidrug resistance (MDR) profile, i.e., TSKQG. Similar findings were obtained with *C. coli* ST-1101, for which the two MDR profiles of the isolates from the flies—i.e., TSEKQG and TSKQG—were not encountered among ST-1101 isolates from feces, which instead exhibited a different MDR profile, TEKQG. Such findings suggest the potentially higher fitness of specific genotype-AMR profile combinations (e.g., *C. jejuni* ST-1839 TSKQG; *C. coli* ST-1101 TSEKQG, TSKQG, and TEKQG) in flies vs. feces.

Whole genome sequencing of a strain of *C. jejuni* ST-1839 from turkey feces and a strain of *C. coli* ST-1067 from a fly in the same turkey house has revealed the genetic basis for similar multidrug-resistance phenotypes in turkeys and flies [52]. Both strains harbored plasmids containing *tet*(O), which confers resistance to tetracycline, as well as a kanamycin resistance gene, together with chromosomal determinants for resistance to nalidixic acid/ciprofloxacin, streptomycin, kanamycin, and gentamicin [52]. Resistance to erythromycin in the *C. coli* strain was conferred by an A2075G substitution in all three copies of the 23S rRNA gene, while the Thr-86-Ile change in *gyrA* in both strains conferred resistance to ciprofloxacin and nalidixic acid [52].

The findings from the current study support the increasing evidence of the importance of flies both as vectors and sentinels for bacterial pathogens and associated AMR determinants [53]. It is of note that multidrug resistant *C. jejuni* of ST-1839, which as noted has previously been frequently reported from turkeys in North Carolina [34,35,46], has now been repeatedly encountered not only in turkey feces but also among multiple fly-derived isolates from the turkey farms. ST-1839 has also been identified in two human clinical samples in the *Campylobacter* MLST database, and among occasional isolates from chicken (https://pubmlst.org/bigsdb?db=pubmlst_campylobacter_isolates&page=profiles; accessed 11 January 2023). It is also interesting to note the shared STs between feces and fly-derived isolates. Indeed, the results of this study indicate that flies from commercial turkey farms are often positive for *Campylobacter*, and may be important in the dissemination of this pathogen as well as associated antimicrobial resistance genes.

## Figures and Tables

**Table 1:** Distribution of isolates by source, species, and year.

Year						
	2013		2014		2015	
Source	*C. coli*	*C. jejuni*	*C. coli*	*C. jejuni*	*C. coli*	*C. jejuni*
Flies	41	51	45	26	1	0
Feces	52	77	43	34	10	0

## Data Availability

Data for this manuscript can be found in the Supplementary Materials. Additional information regarding novel or existing MLST alleles and sequence types can be obtained from PubMLST (https://pubmlst.org/).

## Supplementary Materials

The following supporting information can be downloaded at: https://www.mdpi.com/article/10.3390/pathogens12020230/s1. Table S1: *Campylobacter* Isolates evaluated for multilocus sequence typing and included in Figure 2.
