# Supplementary material for: Campylobacter jejuni and Campylobacter coli from Houseflies in Commercial Turkey Farms Are Frequently Resistant to Multiple Antimicrobials and Exhibit Pronounced Genotypic Diversity
Source: Pathogens. 2023 Feb 1;12(2):230. doi: 10.3390/pathogens12020230 (PMC9965530; doi:10.3390/pathogens12020230)
Supplement: Supplementary file 1 [file pathogens-12-00230-s001.zip › pathogens-2126534-supplementary.pdf]

Table S1: Campylobacter isolates evaluated for Multilocus Sequence Typing and included in Figure 2.

Isolate	ST	CC	Source	Species	Flock	AMR
1	1833	1150	fly	coli	1	TKG
2	1839	--	fecal	jejuni	1	TSKQG
3	1839	--	fly	jejuni	1	TSKQ
4	1839	--	fly	jejuni	1	TKQG
5	1839	--	fly	jejuni	1	TKQ
6	1839	--	fly	jejuni	1	TSKQ
7	1839	--	fly	jejuni	1	TKQ
8	1839	--	fly	jejuni	1	TKG
9	1839	--	fly	jejuni	1	TSKQG
10	2869	828	fly	coli	1	TS
11	9260	1150	fly	coli	1	TKQ
12	1101	828	fecal	coli	2	TSEKQG
13	1101	828	fly	coli	2	TSKQG
14	1101	828	fly	coli	2	TSEKQG
15	1839	--	fecal	jejuni	2	TSKQG
16	2934	353	fecal	jejuni	2	TKG
17	2934	353	fly	jejuni	2	TK
18	2934	353	fly	jejuni	2	TKG
19	1833	1150	fly	coli	3	TKG
20	1833	1150	fecal	coli	3	TKG
21	1839	--	fly	jejuni	3	TSKQG
22	1839	--	fecal	jejuni	3	TSKQG
23	1839	--	fecal	jejuni	3	TSKQG
24	8551	828	fly	coli	3	TSEKQG
25	1833	1150	fecal	coli	4	TEK
26	1833	1150	fecal	coli	4	TKG
27	1839	--	fecal	jejuni	4	TSKQG
28	1192	1150	fly	coli	5	TK
29	1192	1150	fecal	coli	5	TK
30	1833	1150	fecal	coli	5	T
31	1833	1150	fecal	coli	5	T
32	1833	1150	fecal	coli	5	T
33	1839	--	fecal	jejuni	5	TSKQ
34	1839	--	fecal	jejuni	5	TSKQG
35	1067	828	fly	coli	6	TSEKQG
36	1839	--	fly	jejuni	6	TSKQG
37	1839	--	fecal	jejuni	6	TKQ
38	1839	--	fecal	jejuni	6	TSKQG
39	2934	353	fecal	jejuni	6	TKG
40	8213	1150	fly	coli	6	TKG
41	8213	1150	fecal	coli	6	TKQG
42	8213	1150	fecal	coli	6	TKQG

43	8224	1150	fly	coli	6	TKQG
44	8227	--	fly	jejuni	6	TKQ
45	1839	--	fly	jejuni	7	TSKQG
46	1839	--	fecal	jejuni	7	TSKQG
47	1067	828	fly	coli	8	TSEKQG
48	1839	--	fecal	jejuni	8	TSKQG
49	1192	1150	fly	coli	9	TEKQG
50	1192	1150	fly	coli	9	TKQG
51	1839	--	fly	jejuni	9	TSKQG
52	2934	353	fly	jejuni	9	T
53	7730	--	fecal	jejuni	9	TSKQG
54	1101	828	fly	coli	10	TEKQG
55	1101	828	fecal	coli	10	TEKQG
56	1101	828	fecal	coli	10	TEKQG
57	889	828	fecal	coli	11	TSEKQG
58	1017	828	fly	coli	11	TSEKQG
59	1067	828	fly	coli	11	TSEKQG
60	1833	1150	fecal	coli	11	TKG
61	1833	1150	fecal	coli	11	TKG
62	1833	1150	fly	coli	11	TKG
63	9193	1150	fecal	coli	12	TKQG
64	1192	1150	fecal	coli	13	TKQG
65	8224	1150	fecal	coli	14	TSKQG
66	2934	353	fecal	jejuni	15	TKQG
67	2936	--	fecal	jejuni	16	TKQG
68	1161	1150	fecal	coli	17	TSKQG
69	7728	828	fecal	coli	18	SEKQG
